# Optimized inducible shRNA and CRISPR/Cas9 platforms for *in vitro* studies of human development using hPSCs

**DOI:** 10.1242/dev.138081

**Published:** 2016-12-01

**Authors:** Alessandro Bertero, Matthias Pawlowski, Daniel Ortmann, Kirsten Snijders, Loukia Yiangou, Miguel Cardoso de Brito, Stephanie Brown, William G. Bernard, James D. Cooper, Elisa Giacomelli, Laure Gambardella, Nicholas R. F. Hannan, Dharini Iyer, Fotios Sampaziotis, Felipe Serrano, Mariëlle C. F. Zonneveld, Sanjay Sinha, Mark Kotter, Ludovic Vallier

**Affiliations:** 1 Wellcome Trust-MRC Stem Cell Institute, Anne McLaren Laboratory, University of Cambridge, Cambridge, CB2 0SZ, UK; 2 Department of Surgery, University of Cambridge, Cambridge, CB2 0QQ, UK; 3 Department of Clinical Neuroscience, University of Cambridge, Cambridge, CB2 0QQ, UK; 4 Division of Cardiovascular Medicine, University of Cambridge, Cambridge, UK; 5 Wellcome Trust Sanger Institute, Hinxton, CB10 1SA, UK

**Keywords:** Human pluripotent stem cells, shRNA, CRISPR/Cas9, OCT4, POU5F1, T, brachyury, DPY30

## Abstract

Inducible loss of gene function experiments are necessary to uncover mechanisms underlying development, physiology and disease. However, current methods are complex, lack robustness and do not work in multiple cell types. Here we address these limitations by developing single-step optimized inducible gene knockdown or knockout (sOPTiKD or sOPTiKO) platforms. These are based on genetic engineering of human genomic safe harbors combined with an improved tetracycline-inducible system and CRISPR/Cas9 technology. We exemplify the efficacy of these methods in human pluripotent stem cells (hPSCs), and show that generation of sOPTiKD/KO hPSCs is simple, rapid and allows tightly controlled individual or multiplexed gene knockdown or knockout in hPSCs and in a wide variety of differentiated cells. Finally, we illustrate the general applicability of this approach by investigating the function of transcription factors (*OCT4* and *T*), cell cycle regulators (cyclin D family members) and epigenetic modifiers (*DPY30*). Overall, sOPTiKD and sOPTiKO provide a unique opportunity for functional analyses in multiple cell types relevant for the study of human development.

## Abstract (precis)

**Highlighted article:** Novel optimized inducible knockdown and knockout platforms are developed and used to assess gene function in human pluripotent stem cells and their differentiated progeny.

## INTRODUCTION

Loss-of-function experiments in human pluripotent stem cells [hPSCs; comprising human embryonic stem cells (hESCs) or human induced pluripotent stem cells (hiPSCs)] provide a unique opportunity to study the mechanisms that regulate human development, physiology and disease (Avior et al., 2016; Pourquié et al., 2015; Zhu and Huangfu, 2013). However, functional genomic applications of hPSCs are currently limited by the lack of an easy and efficient method to conditionally manipulate gene expression in both hPSCs and hPSC-derived cells. Indeed, such a system is necessary both for the study of genes essential for hPSC self-renewal and for functional analyses at specific stages of differentiation.

Historically, the expression of inducible short hairpin RNAs (shRNAs) has been the most popular method to trigger gene knockdown in human cells. This has been achieved using a TET-ON system, which relies on a modified RNA polymerase (Pol) III promoter that is responsive to a tetracycline-sensitive repressor protein (tetR) to induce shRNA expression by simple tetracycline (TET) treatment (Lambeth and Smith, 2013). Nevertheless, application of this TET-ON system in hPSCs has proved challenging for two main reasons: (1) tight control of shRNA expression is difficult to achieve, thereby resulting in uncontrolled knockdown; (2) induction of shRNA rarely works in differentiated derivatives. Indeed, very high and homogenous expression of both the tetR and the inducible shRNA is required to obtain potent yet controlled knockdown. However, transgene silencing is a recurring problem in hPSCs (Ellis, 2005; Herbst et al., 2012; Yao et al., 2004), and randomly integrated promoters are often subject to positional effects that can strongly limit their activity (Zafarana et al., 2009). Differentiation further increases the chances of silencing, as transgenes can be located in regions where heterochromatin forms following cell fate choices (Herbst et al., 2012; Raya et al., 2009). As a consequence, inducible shRNA expression in both hPSCs and a wide variety of their differentiated progenies has never been reported.

More recently, CRISPR/Cas9-mediated gene knockout has emerged as a powerful method to interrogate gene function (Wright et al., 2016), and inducible manipulation of gene expression in hPSCs using this approach has been reported (Chen et al., 2015; González et al., 2014; Mandegar et al., 2016). However, these methods are either very complex and time consuming, as they involve multiple genome editing steps that need to be individually tailored for each gene to be examined (Chen et al., 2015), or are not widely applicable in multiple differentiated cell types as they rely on inducible promoters that are not stably and homogeneously expressed following hPSC differentiation (González et al., 2014; Haenebalcke et al., 2013; Mandegar et al., 2016; Ordovas et al., 2015). Overall, there are currently no methods for inducible gene knockout in hPSCs that fulfill all the criteria described above.

Here we describe novel platforms for single-step optimized inducible gene knockdown or knockout (sOPTiKD or sOPTiKO) that address all the limitations of current inducible shRNA or CRISPR/Cas9 systems, thus providing powerful and scalable platforms that have the potential to greatly simplify the study of human gene function.

## RESULTS

### Validation of the *ROSA26* and *AAVS1* loci as genomic safe harbors in hPSCs and their differentiated derivatives

**Fig. 1.**
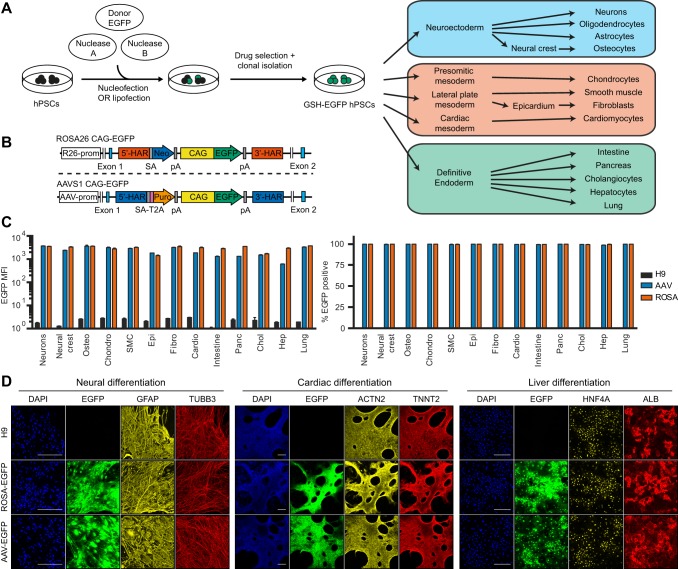
**Validation of the *ROSA26* and *AAVS1* loci as bona fide genomic safe harbors.** (A) Experimental approach behind the generation of genomic safe harbor (GSH) EGFP reporter hPSCs to test GSH expression during differentiation. Neurons, oligodendrocytes and astrocytes were obtained in bulk cultures containing a mixture of these cell lineages, whereas all other cell types were individually generated. (B) *ROSA26* and *AAVS1* EGFP reporter transgenic alleles. R26-prom, *ROSA26* locus promoter; AAV-prom, *AAVS1* locus promoter; 5′-HAR/3′-HAR, upstream/downstream homology arm; SA, splice acceptor; T2A, self-cleaving T2A peptide; Neo, neomycin resistance; Puro, puromycin resistance; pA, polyadenylation signal; CAG, CAG promoter. (C) Summary of EGFP flow cytometry quantification experiments in the indicated cell types generated from GSH EGFP reporter hPSCs (abbreviations indicate the lineages described in A). The percentage of EGFP-positive cells and the EGFP median fluorescence intensity (MFI) are reported. Wild-type hESCs (H9) were used as negative controls, and results are from two independent cultures per lineage. (D) Representative immunofluorescent stainings for lineage-specific markers in three of the mature cell types analyzed. EGFP fluorescence from the reporter lines is in green, and DAPI (blue) shows nuclear staining. Scale bars: 200 μm.

We aimed to develop optimal conditional loss-of-function platforms using inducible shRNAs or guide RNAs (gRNAs) for CRISPR/Cas9. We reasoned that inserting each element of the TET-ON system into a different genomic safe harbor (GSH; Sadelain et al., 2012) would maximize expression in hPSCs and their differentiated progenies while avoiding potential promoter interference (Shearwin et al., 2005). The *AAVS1* and *ROSA26* loci appeared particularly suitable for this purpose as these GSHs have been suggested to allow strong expression of various transgenes in hPSCs, including constitutively expressed shRNAs (DeKelver et al., 2010; Hockemeyer et al., 2009; Irion et al., 2007). We first improved the targeting efficiency for both GSHs by developing a CRISPR/Cas9n-based gene-trap strategy to target the human *ROSA26* locus (Fig. 1A,B, Fig. S1A) and by refining an existing zinc-finger nuclease (ZFN)-based targeting strategy for the *AAVS1* locus (Hockemeyer et al., 2009) (Fig. 1A,B). In both cases, hPSC targeting occurred with very high efficiency (59-100%; Table S1), while neither *ROSA26* nor *AAVS1* modifications resulted in chromosomal abnormalities (data not shown).

We then sought to identify the most efficient promoter to drive constitutive transgene expression from GSHs. We tested the ability of different promoter configurations to express an enhanced green fluorescent protein (EGFP) transgene from the *ROSA26* locus in hESCs (Fig. S1A,B). The highest and most homogenous EGFP expression (100%) was achieved with the artificial CAG promoter (Fig. S1C-E), which was stronger by an order of magnitude than the endogenous *ROSA26* promoter (Irion et al., 2007). Interestingly, and in contrast to previous reports (Ramachandra et al., 2011), we observed that the *EF1α* (*EEF1A1*) promoter was strongly silenced, as shown by mosaic EGFP expression (Fig. S1C-E). Similar results were obtained after EGFP targeting into the *AAVS1* locus (data not shown), thereby preventing the use of this promoter in subsequent experiments.

To further evaluate the robustness of the CAG promoter activity, we analyzed in detail hESCs with heterozygous or homozygous targeting of a CAG-EGFP transgene in the *ROSA26* or *AAVS1* loci (Fig. 1A,B). For both GSHs EGFP was homogeneously expressed at high and comparable levels for more than 30 passages (Fig. S1F), and similar results were obtained after differentiation of hESCs into the three primary germ layers (Fig. S1G-M). Importantly, targeting did not interfere with pluripotency or differentiation, as shown by appropriate expression of lineage markers (Fig. S1N,O). We further differentiated these EGFP-hESC lines into fifteen different cell types (Fig. 1A), and both GSHs allowed homogeneous and strong EGFP expression in all cell types analyzed (Fig. 1C,D, Fig. S2). Overall, these results validate the *ROSA26* and *AAVS1* loci as suitable for robust transgene expression in both hPSCs and their derivatives.

### Development of an optimized inducible knockdown platform in hPSCs

**Fig. 2.**
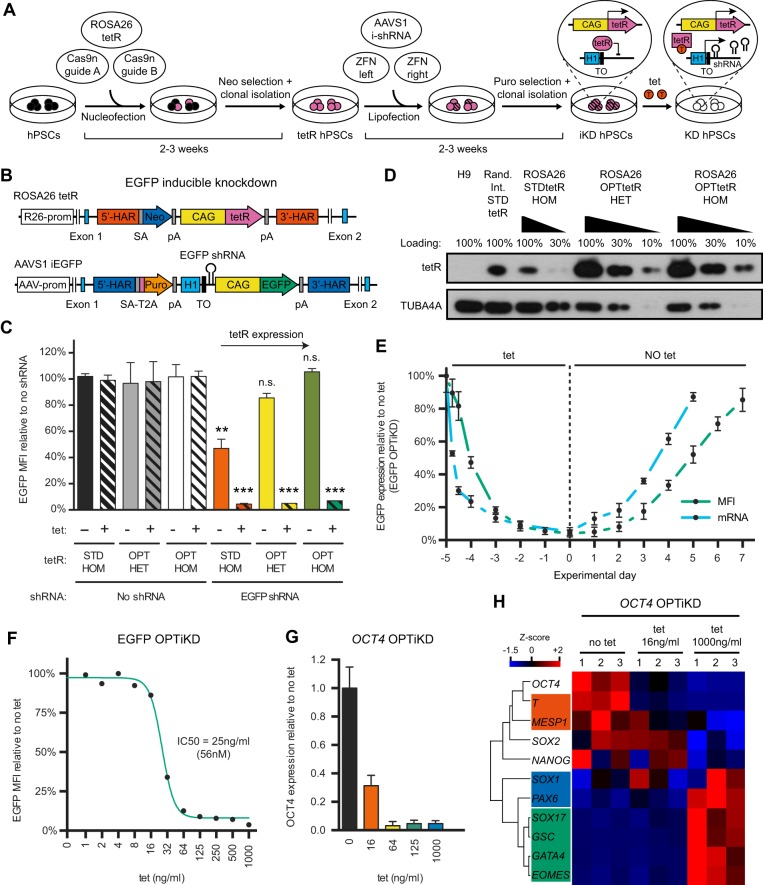
**Development of an optimized inducible knockdown system (OPTiKD) based on dual GSH targeting of hPSCs.** (A) Experimental approach for the generation of inducible knockdown (iKD) hPSCs. H1, H1 promoter; TO, tet operon; tetR, tetracycline-controlled repressor; ZFN, zinc-finger nuclease. (B) Transgenic alleles generated to obtain hESCs expressing an EGFP reporter transgene that could be silenced using an inducible EGFP shRNA. (C) EGFP expression in the absence or presence of tetracycline for 5 days in hESCs targeted with the indicated combinations of inducible EGFP shRNA and tetR [wild-type standard tetR (STDtetR) or codon-optimized tetR (OPTtetR)]. Double-targeted hESCs that did not carry the EGFP shRNA were used as negative controls. Results are from two or three individual lines per condition (see Table S1). n.s., *P*>0.05 (non-significant), **P<0.01, ***P<0.001 versus the same tetR line no tet and no shRNA (ANOVA with post-hoc Holm-Sidak comparisons). (D) Representative western blot for tetR in *ROSA26*-targeted hESCs expressing STDtetR or OPTtetR. HET, heterozygous targeting; HOM, homozygous targeting. hESCs with STDtetR random integration (Rand. Int.) are shown as a positive reference, while wild-type H9 hESCs are negative controls. Various amounts of protein were loaded to facilitate semi-quantitative comparison. TUBA4A (α-tubulin) provided a loading control. (E) EGFP knockdown and rescue kinetics in EGFP OPTiKD hESCs measured by flow cytometry (MFI) and qPCR (mRNA). Results are from two independent cultures per time point. (F) Tetracycline dose-response curve for EGFP knockdown in EGFP OPTiKD hESCs. The half-maximal inhibitory concentration (IC50) is reported. Results are from two independent cultures per dose, and the mean is shown. (G,H) qPCR analysis of *OCT4* OPTiKD hESCs in the absence of tetracycline, or following treatment with different doses of tetracycline for 5 days. (H) Genes are clustered by complete Euclidean distance, and genes specific for pluripotency or for the primary germ layers are in color-coded boxes: no color, hPSCs; red, mesoderm; green, endoderm; blue, neuroectoderm. Z-scores indicate differential expression measured in the number of standard deviations from the average level. Results are from three independent cultures.

Having demonstrated the suitability of the *ROSA26* and *AAVS1* loci for transgene expression, we developed a TET-ON inducible knockdown system based on dual GSH targeting (Fig. 2A, Fig. S3A). To simplify knockdown evaluation and method optimization we generated hESC lines in which an EGFP transgene could be silenced in an inducible fashion (Fig. 2B). To achieve this we targeted: (1) a CAG-tetR expression cassette into the *ROSA26* locus; and (2) a CAG-EGFP transgene plus an inducible EGFP shRNA cassette into the *AAVS1* locus (Fig. 2A,B). Interestingly, we observed a strong and homogeneous decrease in EGFP fluorescence following tetracycline treatment for 5 days (>95%; Fig. 2C), thereby confirming efficient knockdown. However, a decrease in EGFP expression was also noticed in the absence of tetracycline (Fig. 2C), suggesting a significant leakiness in the expression of the shRNA and thus confirming previous reports (Henriksen et al., 2007).

We then hypothesized that this limitation could be bypassed by expressing higher levels of the tetR protein to more strongly repress shRNA expression in the absence of tetracycline. We performed a multi-parameter RNA and codon optimization of the bacterial tetR cDNA (Fath et al., 2011) and used the resulting codon-optimized tetR (OPTtetR) to generate new EGFP inducible knockdown hESC lines (Fig. 2B). This modification achieved a tenfold increase in tetR expression compared with the standard sequence (STDtetR; Fig. 2D). Furthermore, homozygous expression of OPTtetR was sufficient to completely prevent shRNA leakiness while fully preserving efficient knockdown induction (Fig. 2C, Fig. S3B). Of note, the inducible knockdown was rapid, reversible and dose responsive (Fig. 2E,F, Fig. S3C-E). Finally, inducible hESCs displayed a normal karyotype (data not shown), demonstrating that the genome engineering necessary to create these lines did not alter their genetic stability.

Based on these encouraging results, we further validated this method in the context of endogenous genes by generating hESCs carrying inducible shRNAs against *OCT4* (*POU5F1*) or *B2M* (Fig. S3F). Remarkably, all the sublines analyzed (six for each gene) showed robust inducible knockdown with no significant shRNA leakiness (Fig. S3G,H). Tetracycline titration identified optimal concentrations to partially or fully knockdown *OCT4* (Fig. 2G, Fig. S3I,J). As expected, a strong decrease in *OCT4* specifically resulted in loss of pluripotency and induction of neuroectoderm and definitive endoderm markers (Fig. 2H, Fig. S3I,J) (Thomson et al., 2011; Wang et al., 2012). Similar results were obtained with 20 additional *OCT4* inducible knockdown hESC sublines, confirming the robustness and reproducibility of this method (Fig. S3K). Importantly, the generation of hESCs with strong and tightly regulated knockdown was so efficient that phenotypic analyses could be performed immediately after antibiotic selection on a mixed population of cells, thereby entirely bypassing the need to pick individual colonies for clonal isolation (Fig. S3K).

Overall, these results establish that dual targeting of GSHs with an optimized inducible knockdown system is a powerful method to control gene expression in hPSCs. This approach is hereafter named optimized inducible knockdown, or OPTiKD (Fig. 2A, Fig. S3F).

### Single-step generation of optimized inducible knockdown hPSCs

**Fig. 3.**
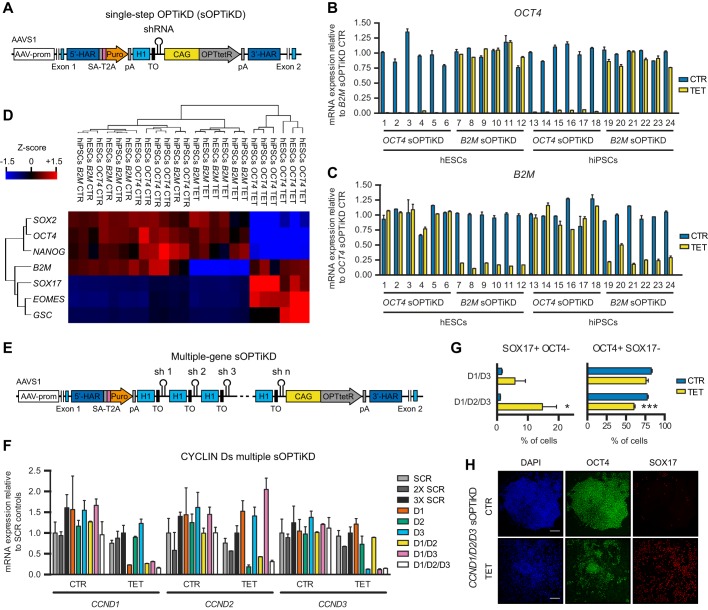
**Single-step optimized inducible knockdown (sOPTiKD) of individual and multiple genes in hESCs and hiPSCs.** (A) The transgenic allele behind the single-step generation of OPTiKD hPSCs. (B,C) qPCR of *OCT4* and *B2M* sOPTiKD hESCs and hiPSCs in the absence (CTR) or presence of tetracycline for 5 days (TET). Individual clonal lines were analyzed in duplicate. (D) Heatmap summarizing qPCR analysis of cells treated as in B,C. Results are from three clonal lines per condition. Samples and genes were clustered by complete Euclidean distance, and Z-scores indicate differential expression measured in number of standard deviations from the average level. (E) The transgenic allele behind the generation of hPSCs with inducible knockdown of multiple genes. (F) qPCR analysis of sOPTiKD hESCs for individual or multiple cyclin D genes (D1, D2 and D3 in the key indicating inducible shRNAs against *CCND1*, *CCND2* and *CCND3*, respectively). Cells were analyzed in the absence or presence of tetracycline for 10 days, and sOPTiKD hESCs carrying one, two or three copies of a scrambled shRNA (SCR, 2X SCR and 3X SCR, respectively) were used as negative controls. For each condition, results are from two clonal pools obtained after gene targeting. (G,H) Flow cytometry quantifications (G) and representative immunostainings (H) for the pluripotency marker OCT4 and the definitive endoderm marker SOX17 in cells treated as in F. *P<0.05, ***P<0.001 versus CTR in the same line (ANOVA with post-hoc Holm-Sidak comparisons). DAPI shows nuclear staining. Scale bars: 100 μm.

We then sought to further improve the OPTiKD system by developing an all-in-one targeting approach that would facilitate the rapid and scalable generation of inducible knockdown hPSCs. We constructed a single *AAVS1* targeting vector to carry both the inducible shRNA and the CAG-tetR expression cassette (Fig. 3A), and validated this approach by knocking down the expression of an EGFP transgene targeted in the *ROSA26* locus (Fig. S3L). Remarkably, this method shared key properties with OPTiKD, such as both the absence of shRNA leakiness (Fig. S3L,M) and rapid, reversible and dose-responsive inducible knockdown (Fig. S3N,O). Thus, this all-in-one strategy, which we named single-step optimized inducible knockdown, or sOPTiKD (Fig. 3A), is as efficient as our original dual targeting approach.

To further demonstrate the versatility of sOPTiKD, we generated both hESC and hiPSC lines carrying an inducible shRNA against *OCT4* or *B2M*. Generation of sOPTiKD hPSCs following lipofection was rapid (2 weeks) and extremely efficient, as all the sublines generated showed robust inducible knockdown (Fig. 3B,C). qPCR analyses confirmed that knockdown of *OCT4* using sOPTiKD induced differentiation of both hESCs and hiPSCs, whereas knockdown of *B2M* had no effect (Fig. 3D). Overall, these experiments show that sOPTiKD provides an efficient system to knock down gene expression that can be easily applied to a large number of hPSC lines.

Finally, we explored whether sOPTiKD could enable simultaneous knockdown of multiple genes (Fig. 3E). We focused on the cyclin D family (*CCND1*, *CCND2* and *CCND3*). These cell cycle regulators are functionally redundant, and thus their study in hESCs has previously required laborious multiple rounds of stable shRNA transfection in order to achieve double or triple knockdown (Pauklin and Vallier, 2013). We developed a method to easily combine multiple shRNAs into the same targeting vector using a one-step Gibson assembly, and generated sOPTiKD plasmids carrying one, two or three shRNAs against cyclin D genes or scrambled control shRNAs (Fig. 3E). These vectors were tested in hESCs without isolation of clonal sublines, and inducible knockdown proved highly efficient and comparable with single, double and triple shRNA constructs (Fig. 3F). Interestingly, prolonged knockdown of one or two cyclin Ds was compatible with hESC self-renewal (Fig. 3G), whereas triple knockdown resulted in definitive endoderm differentiation (Fig. 3G,H), as previously reported (Pauklin and Vallier, 2013; Pauklin et al., 2016). Collectively, these results demonstrate that sOPTiKD can be used to simultaneously decrease the expression of several genes with redundant functions.

### Validation of the optimized inducible knockdown platforms in differentiated progenies of hPSCs

**Fig. 4.**
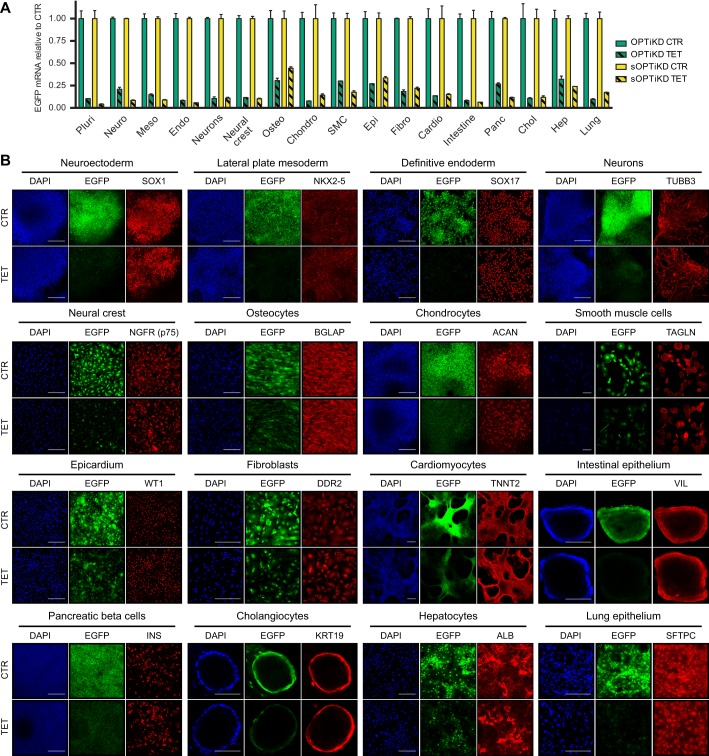
**Validation of the optimized inducible knockdown platforms following hPSC differentiation.** (A) EGFP expression measured by qPCR in the absence (CTR) or presence of tetracycline for 5 days (TET) in the indicated cell types derived from EGFP OPTiKD and sOPTiKD hESCs. EGFP levels are reported relative to control conditions in the same line for each individual lineage. Abbreviations indicate the lineages described in Fig. 1A (pluri indicates undifferentiated). Results are from two independent cultures per condition. (B) Representative immunofluorescent stainings for lineage-specific markers in the three germ layers and in the indicated mature cell types derived from EGFP sOPTiKD hESCs and treated as in A. EGFP fluorescence is in green, and DAPI shows nuclear staining. Similar results supporting the same conclusions were obtained for EGFP OPTiKD hESCs (data not shown). Scale bars: 100 μm for intestinal epithelium and cholangiocytes; 200 μm for all other lineages.

The capacity to knock down genes in a variety of differentiated cells would represent a significant advance over existing systems for inducible gene knockdown. To thoroughly test this possibility, we analyzed the efficacy of the OPTiKD and sOPTiKD platforms to knock down an EGFP transgene in hPSCs differentiated into the three germ layers, as well as in a panel of 13 fully differentiated cell types (Fig. 1A). For both methods, qPCR analyses demonstrated strong and inducible knockdown of EGFP transcripts in all lineages tested (Fig. 4A). Microscopy observations confirmed a robust decrease in EGFP protein expression (Fig. 4B), and flow cytometry showed a decrease in EGFP fluorescence of more than 70% for most lineages (Fig. S4A-G).

Interestingly, EGFP was less reduced in cell types with slower proliferation rates (Fig. S4A). Since EGFP has an extended half-life of more than 24 h, protein loss upon transcriptional or post-transcriptional inhibition relies heavily upon its gradual dilution following cell division (Li, 1998). Considering the strong decrease in EGFP mRNA, we concluded that residual EGFP fluorescence was likely to be a consequence of the relatively short tetracycline treatment performed to trigger knockdown (5 days). To test this, we induced prolonged EGFP knockdown in postmitotic cardiomyocytes, and we indeed observed a slow but constant decrease in protein expression for up to 20 days, at which point the EGFP was decreased by more than 75% (Fig. S4H,I). To reinforce these observations, we performed similar experiments using a *ROSA26*-targeted EGFP reporter transgene fused to a destabilization domain (EGFPd2; Li et al., 1998), which avoids confounding effects due to the long half-life of standard EGFP (Fig. S4J-M) (Wahlers et al., 2001). Remarkably, EGFPd2 inducible knockdown in cardiomyocytes using the sOPTiKD method resulted in >90% protein knockdown after 5 days of tetracycline treatment (Fig. S4N,O). Considered together, these results establish that OPTiKD and sOPTiKD allow efficient manipulation of gene expression even after differentiation of hPSCs.

### Inducible knockdown of T (brachyury) during mesendoderm differentiation of hPSCs

We then sought to exemplify the use of OPTiKD and sOPTiKD to rapidly and efficiently evaluate endogenous gene function in a variety of cell types and at different stages of hPSC differentiation related to embryonic development. First, we focused on the master developmental regulator T (brachyury), which plays an essential role in mesoderm formation and, in particular, during the development of posterior mesoderm, notochord and somites (Martin, 2015; Papaioannou, 2014). Indeed, mice carrying a heterozygous mutation in *T* exhibit a short tail phenotype, while homozygous mutations are embryonic lethal at around 9.5 dpc (Dobrovolskaia-Zavadskaia, 1927; Gluecksohn-Schoenheimer, 1944). *T* mutants also present severe cardiovascular and placental defects (David et al., 2011; Inman and Downs, 2006; King et al., 1998). Furthermore, *T* was recently shown to specifically regulate mesoderm but not endoderm differentiation in hPSCs (Faial et al., 2015).

**Fig. 5.**
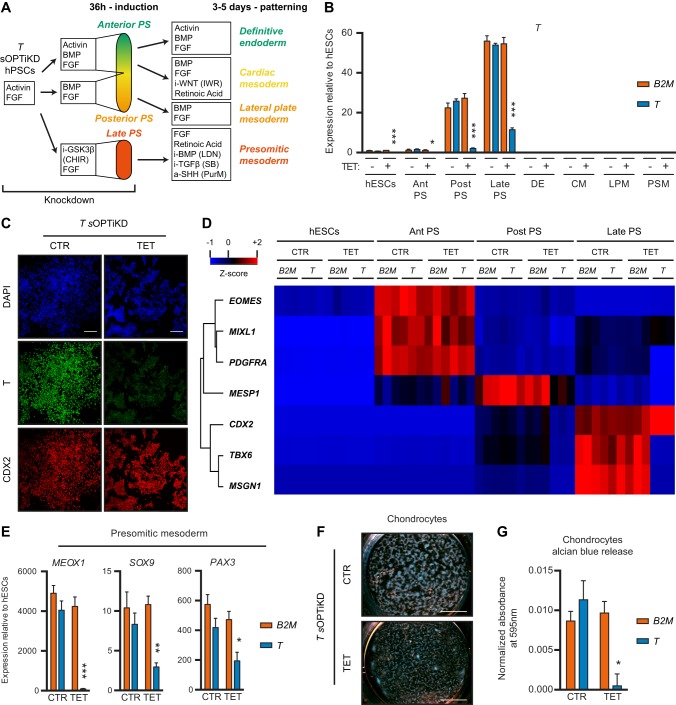
**Functional analysis of T (brachyury) during mesendoderm specification of hPSCs.** (A) The experimental approach. *T* knockdown was induced for 2 days in pluripotent cells and maintained throughout differentiation. PS, primitive streak-like cells; i-, inhibitor; a-, activator; CHIR, CHIR99021; LDN, LDN193189; SB, SB431542; PurM, purmorphamine. (B) qPCR for *T* in the indicated lineages derived from *T* or *B2M* (control) sOPTiKD hESCs as described in A. Ant/Post/Late PS refer to anterior/posterior/late primitive streak; DE, definitive endoderm; CM, cardiac mesoderm; LPM, lateral plate mesoderm; PSM, presomitic mesoderm. (C) Representative immunofluorescent staining demonstrating inducible knockdown of T in late primitive streak cells expressing the marker CDX2. DAPI shows nuclear staining. (D) Heatmap summarizing qPCR results for various mesendoderm markers in primitive streak cells from the experiment described in A,B. Genes were clustered by complete Euclidean distance, and Z-scores indicate differential expression measured in number of standard deviations from the average level. (E) qPCR results for lineage-specific markers in presomitic mesoderm cells from the experiment described in A,B. (F,G) Representative Alcian Blue staining (F) and quantification of Alcian Blue release (G) in chondrocytes differentiated from presomitic mesoderm cells generated as described in A,B. (B,E,G) *P<0.05, **P<0.01, ***P<0.001 versus *B2M* in the same condition (two-way ANOVA with post-hoc Sidak comparisons), and results are from three independent clonal lines per condition. Scale bars: 100 μm in C; 4 mm in F.

To investigate the role of *T* during the differentiation of hPSCs we combined *T* sOPTiKD sublines with culture conditions known to drive the differentiation of hPSCs into subpopulations that recapitulate different portions of the primitive streak and their derived lineages (Fig. 5A) (Bernardo et al., 2011; Cheung et al., 2012; Mendjan et al., 2014; Touboul et al., 2010). Inducible knockdown of *T* was robust in all cell types analyzed (Fig. 5B,C, Fig. S5A,B), confirming the efficiency of sOPTiKD to knock down developmental genes. Decrease in *T* expression did not affect definitive endoderm specification, while differentiation into posterior primitive streak cells, cardiac mesoderm and lateral plate mesoderm was mildly impaired (Fig. 5D, Fig. S5C). By contrast, the generation of late primitive streak progenitors (recapitulating the onset of somitogenesis) and their further specification into presomitic mesoderm and chondrocytes were severely affected following inducible knockdown of *T* (Fig. 5D-G). In particular, induction of *TBX6*, *MSGN1* and *MEOX1* was nearly abolished, in agreement with the established role of *T* in the expression of such genes (Chapman et al., 1996; Faial et al., 2015; Martin, 2015).

Collectively, these results strikingly recapitulate the known role of *T* during early embryonic development, thereby demonstrating the versatility of OPTiKD platforms to study the mechanisms of human development *in vitro*.

### Inducible knockdown of *DPY30* at various stages of hPSC differentiation reveals stage- and lineage-specific functions

**Fig. 6.**
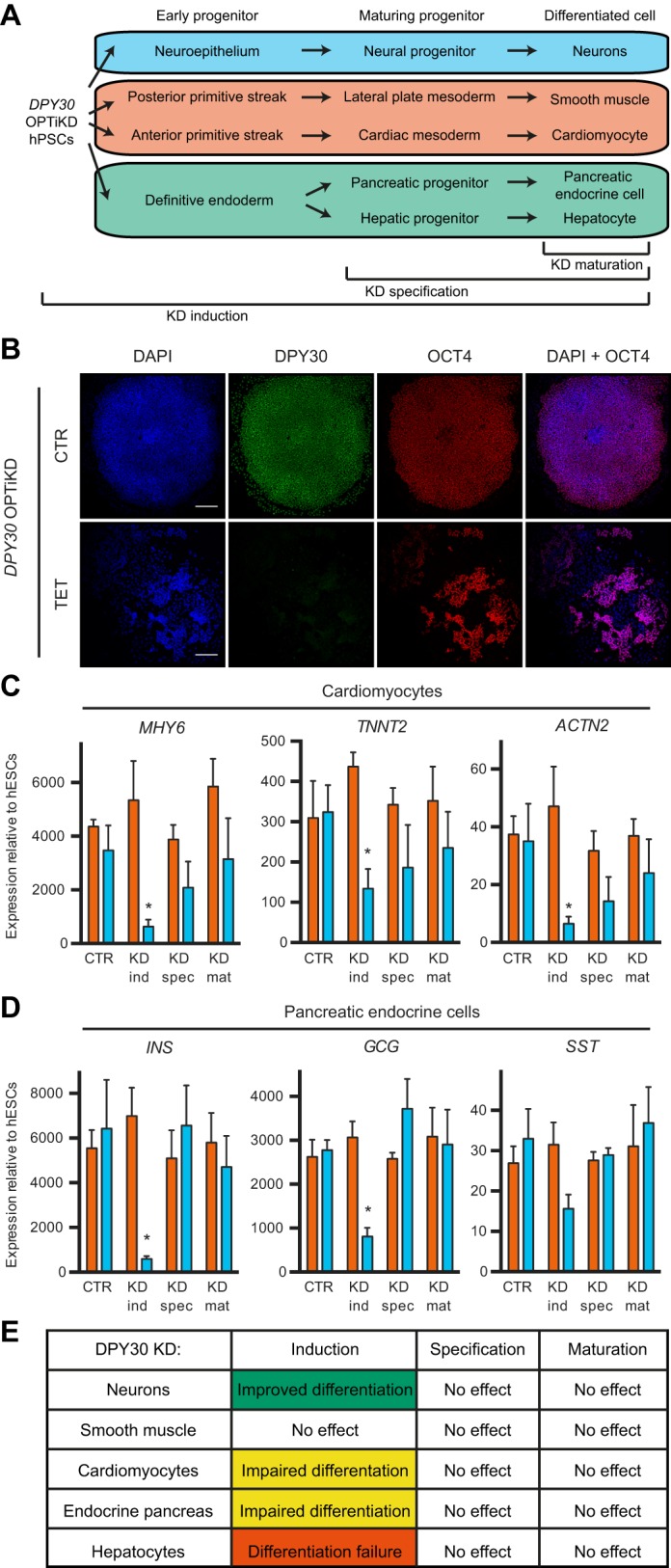
**Functional analysis of *DPY30* during hPSC differentiation.** (A) The experimental design to investigate the role of *DPY30* at various stages of hPSC differentiation. (B) Representative immunofluorescent staining for DPY30 and the pluripotency marker OCT4 in undifferentiated *DPY30* OPTiKD hESCs in the absence (CTR) or presence of tetracycline for 10 days (TET). DAPI shows nuclear staining. Scale bars: 200 μm. (C,D) qPCR-based analyses of *DPY30* (blue) and *B2M* (orange) OPTiKD hESCs after differentiation into mature lineages. CTR, no knockdown; KD ind/spec/mat refer to knockdown from induction/specification/maturation, respectively. Results are from three independent cultures per condition. *P<0.05 versus *B2M* in the same condition (two-way ANOVA with post-hoc Sidak comparisons). (E) Summary of the lineage- and stage-specific phenotypic effects following *DPY30* knockdown during hESC differentiation.

We then aimed to demonstrate the suitability of the OPTiKD platforms to investigate the function of genes that are not only expressed during early development, but also in differentiated cells. We focused on *DPY30*, a ubiquitously expressed co-factor of the COMPASS histone methyltransferase complexes required for histone H3 lysine 4 trimethylation (H3K4me3) (Jiang et al., 2011). This epigenetic modifier is necessary for mouse early embryonic development, as its knockout leads to impaired gastrulation associated with ectopic neuralization of the post-implantation epiblast (Bertero et al., 2015). Similarly, *DPY30* is required for hESC pluripotency (Bertero et al., 2015), and this early role had prevented further studies of its function during differentiation. Finally, *Dpy30* has been implicated in mouse ESC differentiation and in the proliferation and differentiation of hematopoietic progenitors (Jiang et al., 2011; Yang et al., 2014). Consequently, we decided to employ our inducible knockdown platform to bypass the early function of *DPY30* in hPSCs and specifically suppress its expression during differentiation (Fig. 6A).

First, we generated *DPY30* OPTiKD hESC sublines (Fig. S6A), and confirmed that inducible *DPY30* knockdown in undifferentiated hESCs impaired the expression of pluripotency genes and triggered neuroectoderm differentiation (Fig. 6B, Fig. S6B), as shown previously (Bertero et al., 2015). We then analyzed the function of *DPY30* during lineage specification by differentiating *DPY30* OPTiKD hESCs into five different cell types while inducing *DPY30* knockdown from the induction, specification or maturation stages (Fig. 6A). qPCR confirmed the decrease in *DPY30* expression in all the cells generated (Fig. S6C-H). Interestingly, phenotypic analyses demonstrated that *DPY30* knockdown from the early induction of cardiac specification impaired cardiomyocyte differentiation, as shown by the decrease in contractile markers (Fig. 6C). However, knockdown at later stages had no significant effects (Fig. 6C). A similar result was observed for the hepatocyte lineage, since decrease of *DPY30* expression in endoderm progenitors led to extensive cell death at the anterior foregut stage thereby preventing further differentiation (Fig. S6I). Similarly, specification of pancreatic endocrine cells was also impaired by knockdown of *DPY30* in the initial stage of differentiation (Fig. 6D). However, neither hepatocyte nor pancreatic endocrine cell specification was significantly affected by knockdown of *DPY30* in maturing progenitors or differentiated cells (Fig. 6D,E, Fig. S6I). By contrast, neuronal differentiation was promoted following *DPY30* knockdown during the induction of neuroepithelial progenitors (Fig. S6J). Finally, *DPY30* knockdown at any stage during smooth muscle cell differentiation had no effect on the expression of key lineage markers (Fig. S6K).

Considered together, these data confirm a key role for *DPY30* during germ layer specification while suggesting that the requirement for *DPY30* expression could vary during the differentiation and maturation of specific lineages (Fig. 6E). Overall, these experiments illustrate how the optimized inducible knockdown platform can be easily applied to acquire novel information about developmental mechanisms by performing functional studies at different steps of hPSC differentiation into multiple cell types.

### Development of an optimized inducible CRISPR/Cas9 knockout platform in hPSCs

**Fig. 7.**
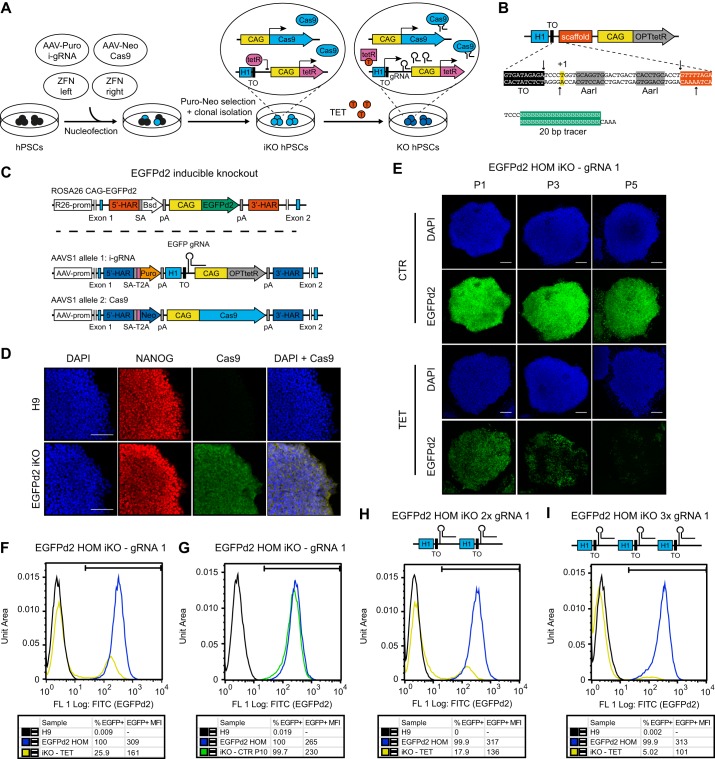
**Development of an optimized inducible CRISPR/Cas9 knockout platform in hPSCs.** (A) Experimental approach for the generation of inducible knockout (iKO) hPSCs. (B) The cloning procedure to generate *AAVS1* targeting vectors with an inducible gRNA cassette. The arrows indicate the DNA cut sites induced by digestion with *Aar*I. (C) Transgenic alleles generated to obtain hESCs expressing an EGFPd2 reporter transgene that could be knocked out by CRISPR/Cas9 using an inducible EGFP gRNA (EGFP sOPTiKO hESCs). Bsd, blasticidin resistance; EGFPd2, destabilized EGFP. (D) Representative immunofluorescent stainings for Cas9 in EGFPd2 homozygous sOPTiKO hESCs. Wild-type hESCs (H9) were analyzed as a negative control. Cells were co-stained for the pluripotency factor NANOG, and DAPI shows nuclear staining. (E) Representative images depicting EGFPd2 fluorescence in EGFPd2 homozygous sOPTiKO hESCs in the absence (CTR) or presence of tetracycline (TET) for the indicated number of passages (P; cells were split every 5 days). (F) Representative flow cytometry for EGFPd2 expression in EGFPd2 homozygous sOPTiKO hESCs (iKO) following three passages in the presence of tetracycline. EGFPd2 homozygous cells that do not carry the inducible CRISPR/Cas9 system (EGFPd2 HOM) and wild-type hESCs were analyzed as positive and negative controls for EGFPd2 expression, respectively. The gate used to define EGFPd2-positive cells (EGFP^+^) is shown, and the percentage of EGFP^+^ cells and their MFI are reported. (G) As in F, but EGFPd2 homozygous sOPTiKO hESCs were analyzed following ten passages in the absence of tetracycline. (H,I) As in F, but EGFPd2 homozygous sOPTiKO hESCs were generated using an *AAVS1* targeting vector carrying two or three copies of the inducible EGFP gRNA cassette (2× and 3× gRNA, respectively). All results in this figure were obtained using EGFP gRNA 1. Scale bars: 100 μm.

Having established an optimized inducible knockdown platform, we turned our attention to developing a complementary inducible knockout approach. Current inducible CRISPR/Cas9 methods rely on conditional overexpression of Cas9 in the presence of a constitutively expressed gRNA (González et al., 2014; Mandegar et al., 2016). In this case, control of Cas9 overexpression is achieved by a TET-ON method in which, following doxycycline treatment, a tetracycline-controlled reverse transactivator (rtTA) activates a Pol II-dependent tetracycline-responsive element (TRE) promoter (a fusion between multiple TET operons and a minimal CMV promoter). Although this TET-ON platform has been successfully applied to certain human cell types (Qin et al., 2010), we observed that this inducible system is silenced during hPSC differentiation into multiple lineages (including cardiomyocytes, hepatocytes and smooth muscle cells), even after targeting into the *AAVS1* GSH (Fig. S7). These observations reinforce previous reports (Haenebalcke et al., 2013; Mandegar et al., 2016; Ordovas et al., 2015) and demonstrate that recently described systems for inducible CRISPR/Cas9 (González et al., 2014; Mandegar et al., 2016) are unlikely to work in a diversity of hPSC-derived cell types. For this reason, we explored the possibility of developing an alternative and improved method by combining a constitutively expressed CAG promoter-driven Cas9 with an inducible gRNA cassette based on that developed for inducible shRNA expression in sOPTiKD (Fig. 7A,B).

We generated hESC lines in which a fluorescent reporter gene could be knocked out in an inducible fashion (Fig. 7C). For this, we targeted *ROSA26*-EGFPd2 reporter hESCs (Fig. S4J,K) with both an inducible EGFP gRNA and a constitutive Cas9 in the *AAVS1* locus, each transgene being integrated into one of the two alleles (Fig. 7C,D). This dual targeting approach was rapid (<2 weeks) and efficient (>90% of lines containing both transgenes; Table S1). Remarkably, when individual clonal sublines were grown in the presence of tetracycline we observed decreased EGFPd2 expression in all of the targeted lines, and EGFPd2 homozygous cells showed near-homogeneous loss of at least one copy of the reporter gene as early as 5 days following tetracycline induction (as demonstrated by 50% reduction in EGFPd2 fluorescence, Fig. S8A). Prolonged treatment with tetracycline progressively led to the complete loss of EGFPd2 fluorescence in up to 75% of EGFPd2 homozygous cells (Fig. 7E,F, Fig. S8A,B). Interestingly, co-expression of either two or three copies of the same EGFP gRNA cassette from the same *AAVS1* locus was sufficient to significantly increase the speed and efficiency of inducible EGFPd2 knockout in all the clonal sublines analyzed (Fig. 7H,I, Fig. S8A). For instance, simultaneous induction of three copies of the same gRNA resulted in a remarkable 95% knockout efficiency following tetracycline treatment (Fig. 7I). Importantly, inducible EGFPd2 knockout hESCs did not show any significant decrease in the proportion of EGFPd2-positive cells nor in their fluorescence after prolonged culture in the absence of tetracycline, even when several gRNA copies were used (Fig. 7G, Fig. S8C,D). This demonstrated that the inducible gRNA expression was tightly controlled. Finally, testing of additional gRNAs against EGFPd2 revealed that the speed and efficiency of the inducible knockout strongly relied on the gRNA. Indeed, an optimal sequence allowed up to 90% knockout after only 2 days of induction (Fig. S8E,F,K). Of note, the most efficient gRNA also resulted in uncontrolled EGFPd2 knockout (Fig. S8G), but this limitation was avoided by simply adding a second TET operon to the inducible H1 promoter to ensure even more stringent transcriptional control (Fig. S8H-K).

Collectively, these results show that the sOPTiKD system could be readily repurposed to support inducible gRNA expression and allow tightly controlled activity of CRISPR/Cas9 over a broad range of gRNA potency (Fig. S8L). To the best of our knowledge, this is the first conditional CRISPR/Cas9 approach based on inducible gRNA expression. We named this method single-step optimized inducible gene knockout, or sOPTiKO.

### Validation of the optimized inducible CRISPR/Cas9 platform in differentiated progenies of hPSCs

**Fig. 8.**
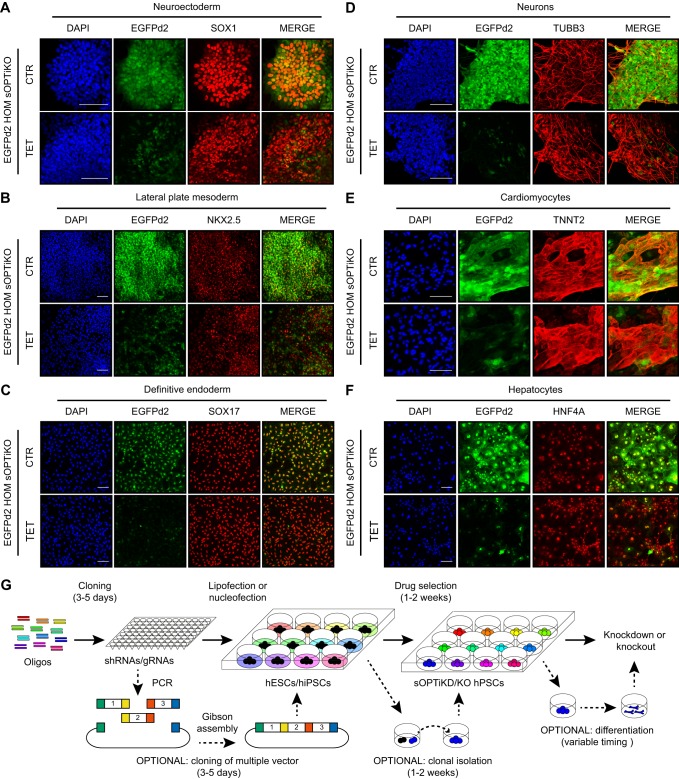
**Validation of the optimized inducible CRISPR/Cas9 platform following hPSC differentiation.** (A-F) Representative immunofluorescent stainings for the indicated lineage-specific markers in cells derived from EGFPd2 sOPTiKO hESCs carrying a single EGFP inducible gRNA (gRNA 1). EGFPd2 knockout was induced with tetracycline for 6 days for the germ layers (A-C) and for 10 days for the mature cells (D-F). EGFPd2 fluorescence in control conditions (CTR) or after knockout (TET) is in green, and DAPI shows nuclear staining. Merged images of the EGFPd2 and lineage-specific markers are shown. Scale bars: 100 μm. (G) Summary of the experimental strategy behind the generation and application of inducible knockdown or knockout hPSCs using the sOPTiKD or sOPTiKO platforms.

Having demonstrated that sOPTiKO allows efficient control of CRISPR/Cas9 activity in undifferentiated hPSCs, we thoroughly tested its performance following differentiation. We differentiated homozygous EGFPd2 inducible knockout cells carrying a single copy of inducible EGFP gRNA into the three primary germ layers and into five cell types of clinical interest (neurons, cardiomyocytes, smooth muscle cells, hepatocytes and endocrine pancreatic cells). Immunostaining for lineage-specific markers demonstrated that treatment with tetracycline resulted in strong loss of EGFPd2 expression (Fig. 8A-F, Fig. S9A,B) in all these cell types. Moreover, flow cytometry quantification confirmed that inducible knockout in differentiated cells was tightly controlled and efficient (Fig. S9C-H). For example, 85% of neuronal cells and 75% of smooth muscle cells completely lost EGFPd2 expression following tetracycline treatment (Fig. S9C,F). Considered together, these results validate that sOPTiKO allows efficient control of CRISPR/Cas9 activity not only in hPSCs, but also into a large panel of mature cell types (Fig. 8G).

## DISCUSSION

This report describes sOPTiKD and sOPTiKO – two novel platforms for inducible knockdown or knockout of gene expression that address the limitations of previous methods. Compared with alternative approaches that rely on viral transduction or random integration of inducible shRNAs (Lambeth and Smith, 2013; Zafarana et al., 2009), sOPTiKD is simpler to use (plasmid based), quicker (2 weeks or less to generate stable lines following a single step of gene targeting by lipofection), more efficient (>95% of the resulting cells show inducible knockdown), more scalable (isolation of clonal sublines can be entirely bypassed) and significantly more robust (due to the use of GSHs and the lack of leakiness). Furthermore, sOPTiKO shares these same advantages, thus outperforming recent inducible CRISPR/Cas9 methods that rely on the conditional expression of Cas9 (González et al., 2014) or of a fusion protein between a catalytically inactive Cas9 and the transcriptional repressor KRAB (Mandegar et al., 2016). Indeed, these systems rely on the TRE promoter, which is heavily silenced upon hPSC differentiation into multiple lineages. Furthermore, these are lengthy two-step methods, and expression of the gRNA is achieved either by transient transfection, which can be poor in efficiency, or by random integration of the gRNA, which can result in mosaic expression. Finally, whereas CRISPR interference can only efficiently control gene promoter activity (Mandegar et al., 2016), sOPTiKO allows the deletion of a broader range of genomic targets, including regions outside of promoters that might not have a direct role in transcriptional regulation. Overall, sOPTiKD/KO are the first inducible shRNA and CRISPR/Cas9 technologies that enable streamlined functional studies of multiple genetic variants in hPSCs and in a diversity of differentiated cell types (Fig. 8G).

sOPTiKD and sOPTiKO each presents distinct advantages. On the one hand, the ability to control the level of knockdown using sOPTiKD allows the study of genes for which complete loss-of-function induces cell death, and facilitates the examination of gene dosage mechanisms. On the other hand, phenotypic studies following full gene knockout using sOPTiKO are more relevant in the case of genes that are still functional even when expressed at low levels. Moreover, sOPTiKO is applicable not only to genes, but also to non-coding genomic regulatory regions, which could represent a majority of disease-associated genetic traits (Cooper and Shendure, 2011).

Aside from the examples reported in this manuscript, we envision several other potential applications of the sOPTiKD/KO technologies. With regard to cellular and developmental biology, we anticipate that sOPTiKO could efficiently accommodate variants of the Cas9 gene with catalytically inactive domains (Dominguez et al., 2015). For instance, Cas9 fusion proteins with epigenetic modifiers could allow functional validations of putative genomic regulatory regions. Similarly, sOPTiKD could be repurposed to drive other types of inducible non-coding RNAs, such as antagomir or miRNA sponges to probe microRNA function (Ebert and Sharp, 2010). Remarkably, the high targeting efficiency and scalability of sOPTiKD/KO could allow high-throughput screenings by targeting inducible shRNA or gRNA pools. Compared with viral-based approaches (Chen et al., 2012), the isogenic integration of inducible shRNAs/gRNAs would reduce heterogeneity in the targeted population, hence increasing the screening sensitivity and specificity. With regard to disease modeling applications, sOPTiKD/KO could allow the simultaneous targeting of several hiPSC lines to probe gene function in different genetic backgrounds. Such an approach could facilitate the identification of genetic disease modifiers and the discovery of novel potential drug targets in the context of personalized medicine. Multiplex inducible gene knockdown or knockout could also be used to model complex genetic disorders. Finally, sOPTiKD/KO could be easily transferred to other cell types amenable to genetic manipulation, including established cell lines and adult stem cells (Drost et al., 2015; Mandal et al., 2014), thus allowing functional studies in a multitude of systems. In conclusion, we expect that sOPTiKD/KO technologies will have a broad impact on our ability to study human development, physiology and disease.

## MATERIALS AND METHODS

### hPSC culture and differentiation

Feeder- and serum-free hESC (H9 line; WiCell) and hiPSC (A1AT^R/R^ line; Rashid et al., 2010) culture and differentiation were as previously described (Vallier, 2011). Details of media compositions and protocols are provided in the supplementary Materials and Methods.

### Gene targeting

Sequences of all plasmids used in this study are provided in Appendix S1, and all cloning procedures and targeting experiments are described in detail in the supplementary Materials and Methods. Briefly, *AAVS1* targeting for OPTiKD and sOPTiKD was performed by lipofection, while *AAVS1* targeting for sOPTiKO was performed by nucleofection (Bertero et al., 2015; Vallier et al., 2004). Clonal lines were selected using 1 μg/ml puromycin (Sigma; for OPTiKD and sOPTiKD) or 25 μg/ml geneticin (G418 sulfate, Gibco) and 0.5 μg/ml puromycin (for sOPTiKO).

### Inducible gene knockdown and knockout

Unless otherwise described in the results or figure legends, tetracycline hydrochloride (Sigma-Aldrich) was used at 1 μg/ml to induce gene knockdown or knockout. Refer to the supplementary Materials and Methods for details on the timing of *DPY30* inducible knockdown during hESC differentiation.

### Analysis of RNA and protein expression

Quantitative real-time PCR (qPCR), western blot, flow cytometry and immunofluorescence were performed according to standard protocols as previously described (Bertero et al., 2015). Details, including the primer sequences and antibodies used, are provided in the supplementary Materials and Methods.

### Statistical analysis

Statistical analyses were performed using GraphPad Prism 6. The type and number of replicates, the statistical test used, and the test results are described in the figure legends. All statistical tests employed were two-tailed. Unless stated otherwise in the figure legends, all graphical data are presented as mean±s.e.m. No experimental samples were excluded from the statistical analyses. Sample size was not pre-determined through power calculations, and no randomization or investigator blinding approaches were implemented during the experiments and data analyses. When representative results are presented, the experiments were reproduced in at least two independent cultures.
